# Distinguishing PFAS biotransformation from defluorination and destruction: a fluorine-fate evidence framework

**DOI:** 10.3389/fsysb.2026.1991575

**Published:** 2026-10-28

**Authors:** Mohd Faheem Khan

**Affiliations:** UCD School of Agriculture and Food Science, University College Dublin, Dublin, Ireland

**Keywords:** biodegradation, biotransformation, C-F bond cleavage, defluorination, fluorine mass balance, microbial degradation, PFAS, transformation products

## 1 Introduction

Per- and polyfluoroalkyl substances (PFAS) are a structurally diverse class of persistent, mobile, and bioaccumulative contaminants, with more than one megatonne produced annually and continuing releases throughout manufacture, use, and disposal (Evich et al., 2022). Volatile PFAS and their precursors can undergo long-range atmospheric transport and transform into persistent terminal PFAS, facilitating their circulation among air, water, soil, sediment, and biota (Renella and Quintarelli, 2026; Evich et al., 2022). Their environmental recalcitrance is strongly associated with the exceptional stability of the C–F bond, for which reported bond dissociation energies depend on the molecular environment but are generally high in highly fluorinated organic compounds (approximately 485 kJ mol^-1^ as a representative value), together with its short bond length, high electronegativity, chemical stability, and limited biological accessibility (Khan, 2025; Renella and Quintarelli, 2026). PFAS are consequently detected from ng L^-1^ to μg L^-1^ across environmental matrices, while contaminated agricultural and industrial soils can remain long-term sources to groundwater and food webs (Khan, 2025; Khan, 2026a; Kareem and Khan, 2026). These physicochemical characteristics underpin PFAS persistence and provide the chemical basis for distinguishing molecular transformation from C–F bond cleavage and the extent of fluorine and carbon conversion associated with destructive treatment (Figure 1A).

**FIGURE 1:**
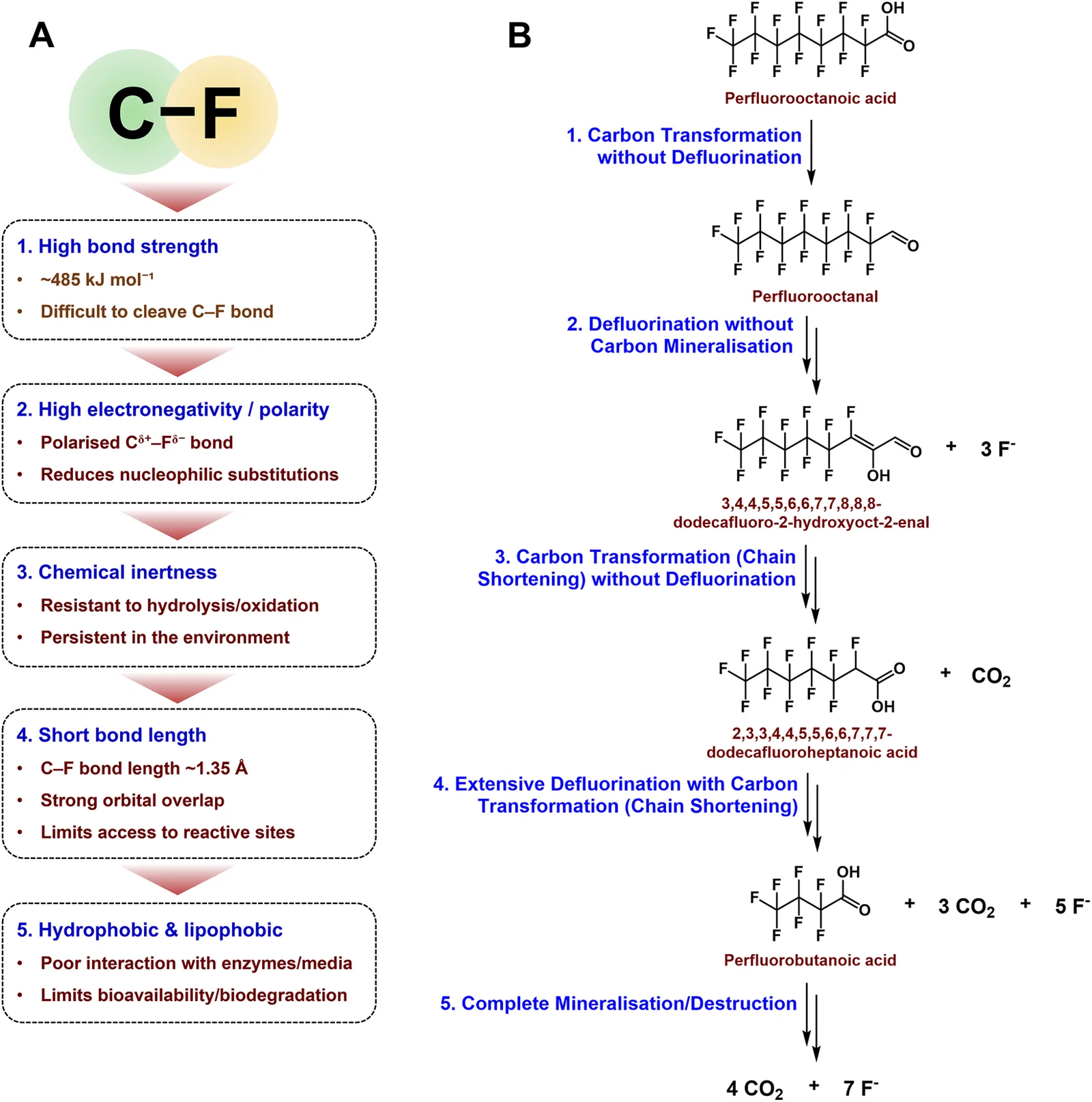
Distinguishing PFAS biotransformation, defluorination, and mineralisation: a conceptual fluorine-fate evidence framework. **(A)** Key physicochemical properties of C–F bonds and fluorinated structures that contribute to PFAS persistence and may limit microbial accessibility and biodegradation. **(B)** Conceptual pathways illustrating distinct outcomes of PFAS transformation: (1) transformation without defluorination; (2) defluorination without complete carbon mineralisation; (3) carbon transformation through chain shortening without complete defluorination; (4) extensive defluorination accompanied by carbon transformation; and (5) a mineralisation-type endpoint involving conversion of organic carbon to CO_2_ and organofluorine-bound fluorine to inorganic fluoride (F^−^). These outcomes are not interchangeable: parent-compound disappearance does not establish defluorination, fluoride release does not demonstrate carbon mineralisation, and carbon transformation does not necessarily indicate complete fluorine removal. Extensive defluorination and carbon mineralisation are distinct endpoints and should be supported by separate lines of evidence. The pathways are illustrative rather than a universal or necessarily sequential degradation route.

This persistence has stimulated extensive research into PFAS remediation, including adsorption, ion exchange, membrane separation, foam fractionation, soil stabilisation, thermal treatment, electrochemical processes, and plasma technologies. Separation and stabilisation can substantially decrease aqueous concentrations or suppress leaching, including reported reductions of approximately 60%–90% following soil stabilisation, but generally transfer or immobilise rather than destroy the fluorinated mass (Uddin et al., 2026). Destructive technologies are therefore increasingly evaluated using defluorination and mineralisation rather than parent-PFAS removal alone (Tshangana et al., 2025; Meegoda et al., 2022; Wen et al., 2026). These non-biological technologies provide useful comparators for evaluating the relationship between parent-PFAS degradation and fluorine removal; they are included here to clarify the evidence required to substantiate destructive remediation, rather than to imply biological mechanisms. Biological approaches have attracted particular interest because bacteria, fungi, algae, consortia, and enzymes can operate under relatively mild conditions, with proposed involvement of fluoroacetate dehalogenases, cytochrome P450s, reductive dehalogenases, oxygenases, hydrolases, peroxidases, and laccases (Liang and Zhao, 2026; Khan et al., 2023a). Metagenomics, CRISPR, multi-omics, metabolic modelling, synthetic biology, and enzyme engineering are further accelerating the search for biological PFAS transformation and biodefluorination pathways (Khan, 2025; Olawade et al., 2025; Che et al., 2025).

The concern is that terminology has often advanced faster than the evidence. Parent-PFAS disappearance can result from adsorption, bioaccumulation, sequestration, precipitation, volatilisation, or phase redistribution, while transformation may simply redistribute fluorine among shorter-chain PFAS, fluorotelomer products, or other unmonitored organofluorines (Cao et al., 2025; Huang et al., 2025). Even genuine molecular transformation therefore does not necessarily constitute destruction. For example, *Labrys portucalensis* F11 removed approximately 90% PFOS and 58% 5:3 FTCA after 100 days, with PFOS removal reaching 96% after 194 days, but only 21% of 6:2 FTS was removed after 100 days. PFOS transformation generated PFHpS, PFHxS, PFHxA, PFPeA, PFBA, PFPrA, and three defluorinated products, whereas 5:3 FTCA produced shorter-chain and unsaturated fluorotelomer products and 6:2 FTS produced 4:2 FTS (Wijayahena et al., 2025). These results demonstrate biological transformation and partial defluorination, but not complete destruction. Accordingly, the evidence needed to characterise PFAS remediation should distinguish several related but non-equivalent outcomes: parent-PFAS disappearance, transformation-product formation, direct evidence of C–F bond cleavage, fluoride release, fluorine mass balance, and carbon mineralisation (Figure 1B). These outcomes form an evidence framework rather than a universal sequence: transformation may occur without demonstrable C–F bond cleavage; defluorination may occur without complete carbon mineralisation; and carbon-chain transformation may occur without complete removal of organofluorine. Importantly, these outcomes should not be treated as interchangeable: biological transformation may occur without demonstrable C–F bond cleavage, whereas biodefluorination specifically requires evidence of fluorine removal from the organic molecular framework. Extensive defluorination and carbon mineralisation should likewise be evaluated as distinct endpoints, because evidence for one does not, by itself, establish the other. This Opinion therefore argues that parent-PFAS disappearance alone cannot establish biodegradation or destructive remediation. Instead, the terminology should reflect the demonstrated outcome, with convincing fluorine-fate evidence required to substantiate claims of biodefluorination and PFAS destruction.

## 2 What should “PFAS biodegradation” actually mean?

A scientifically meaningful definition of PFAS biodegradation should distinguish removal, transformation, defluorination, and mineralisation according to the evidence obtained. Removal indicates a concentration decrease, while sorption or sequestration represents redistribution without destruction (Wackett, 2021; Cao et al., 2025). PFAS biotransformation refers to biological conversion of the parent compound into one or more products, irrespective of whether C-F bond cleavage occurs. Partial biodegradation may involve biological structural modification while retaining substantial organofluorine. Biodefluorination specifically requires evidence of C-F bond cleavage and fluorine removal from the organic molecular framework. Mineralisation involves conversion of organic constituents towards inorganic end products and must be distinguished from defluorination, because fluoride release alone does not demonstrate mineralisation of the carbon skeleton (Wackett, 2021; Wackett and Robinson, 2024). Claims of PFAS destruction should therefore be supported by convincing evidence of defluorination and an assessment of fluorine fate, while claims of mineralisation additionally require evidence of carbon-product conversion and, where feasible, carbon mass balance (Wackett and Robinson, 2024; Wen et al., 2026). Accordingly, claims of mineralisation require evidence of carbon-product conversion and, where feasible, carbon mass balance. These outcomes depend on PFAS structure, redox conditions, oxygen availability, enzyme specificity, and microbial interactions (Skinner et al., 2025; Che et al., 2025; Renella and Quintarelli, 2026).

### 2.1 Transformation is not defluorination

The high stability of the C-F bond makes its biological activation challenging, although microbial C-F cleavage has been documented across fluorinated aliphatic and aromatic compounds and increasingly in PFAS-relevant systems (Natarajan et al., 2005; Liu et al., 2024). The distinction between transformation and defluorination is illustrated by *Cunninghamella elegans*, which completely consumed 100 mg L^-1^ 6:2 FTOH within 48 h while accumulating 5:3 FTCA. The product was identified using ^19^F NMR, GC-MS, and LC-MS, and inhibition by 1-aminobenzotriazole implicated cytochrome P450 activity; 5:3 FTCA itself inhibited P450-mediated metabolism, contributing to its accumulation (Khan and Murphy, 2023). Similarly, individual photocatalytic and fungal treatments of 100 ppm PFOA achieved approximately 35%–40% degradation but only 20%–30% defluorination, whereas sequential photocatalysis-fungal treatment increased degradation to 90% and defluorination to 60%; photocatalysis produced short-chain PFCAs while fungal treatment predominantly produced 5:3 FTCA (Khan et al., 2023b). This combined treatment provides a direct comparison of physicochemical and biological processes, illustrating how complementary treatment steps can improve both transformation and defluorination. These results show that parent-compound conversion and fluorine removal can diverge substantially, even when transformation is extensive.

Precursor transformation provides an additional warning. N-EtFOSAA can biologically transform to PFOS, meaning that disappearance of a precursor can generate rather than eliminate a persistent terminal PFAS (Thapa et al., 2026). Conversely, some structures can undergo transformation that initiates genuine defluorination. Jin et al. (2023) showed that susceptible ether PFAS containing a CH_2_ group adjacent to an ether linkage or a C=C bond can undergo oxidative or hydrolytic O-dealkylation to unstable fluoroalcohols that subsequently defluorinate. Transformation-product identification is therefore essential for resolving PFAS fate, and reaction libraries can support systematic prediction and interpretation of environmental and biological pathways (Weber et al., 2022). These examples show that transformation-product profiles are mechanistically informative, but their implications for fluorine fate must be established independently.

### 2.2 Fluoride release as evidence of C-F bond cleavage

Direct measurement of fluoride provides stronger evidence of C-F bond cleavage because hydrolytic, reductive, and eliminative pathways can release F^−^ alongside organic products (Simon et al., 2025). Yu et al. (2020) demonstrated microbial reductive defluorination of two C_6_ per- and polyfluorinated compounds by an organohalide-respiring community, coupling product formation with direct F^−^ release under lactate-fed conditions; although *Dehalococcoides* was present, it was not responsible for the observed activity. More recent pH-based screening has expanded high-throughput discovery of defluorinases, but proton release remains an indirect indicator compared with direct fluoride quantification (Simon et al., 2025). Fluoride yields should be reported relative to the theoretical fluorine content of the substrate, with corrections for background fluoride, analytical recovery, and substrate stoichiometry.

The fungal transformation of PFOA to 5:3 fluorotelomer carboxylic acid (5:3 FTCA) has been experimentally reported, with 5:3 FTCA identified as the main product of fungal treatment (Khan et al., 2023a). PFOA contains 15 F atoms, whereas 5:3 FTCA contains 11; therefore, a hypothetical 1:1 M conversion corresponds to the potential release of four F atoms per transformed PFOA molecule. Figure 2 uses this experimentally reported transformation as the basis for an illustrative fluorine-fate calculation, assuming 60% conversion. The assumed conversion and calculated fluorine distribution are hypothetical and should not be interpreted as experimentally measured outcomes. The calculation illustrates how parent transformation may retain most substrate fluorine in an organic product; it does not establish that this fluoride yield or product distribution occurred experimentally.

**FIGURE 2:**
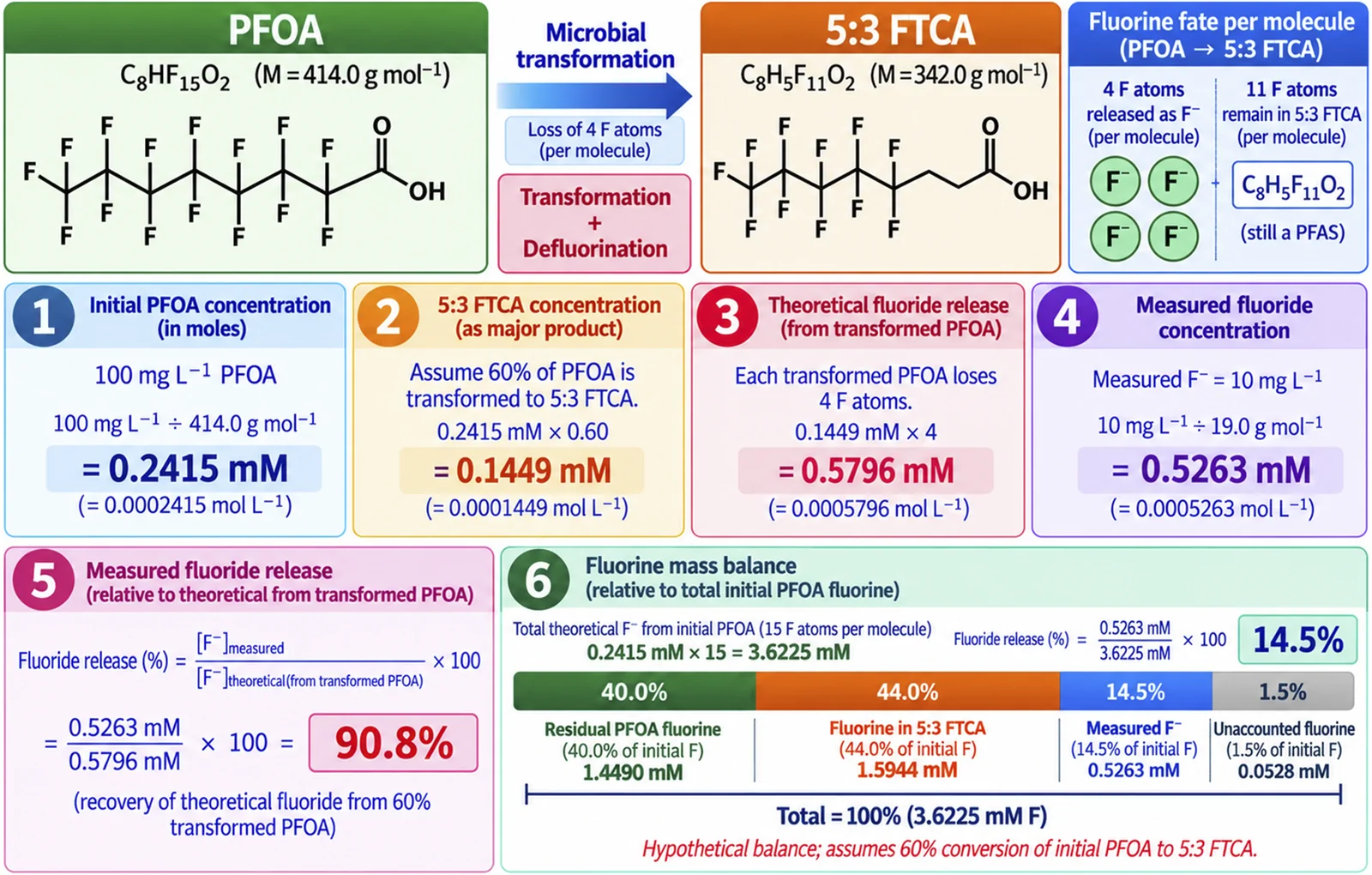
Hypothetical quantitative illustration of fluorine fate during the reported fungal transformation of PFOA to 5:3 fluorotelomer carboxylic acid (5:3 FTCA). PFOA (C_8_HF_15_O_2_; 414.0 g mol^-1^) can be transformed to 5:3 FTCA (C_8_H_5_F_11_O_2_; 342.0 g mol^-1^), which was identified as a major product of fungal PFOA treatment by Khan et al. (2023a). The transformation pathway is experimentally reported; however, all numerical values shown are hypothetical and do not reproduce the experimental results. The calculation assumes an initial PFOA concentration of 100 mg L^-1^, 60% molar conversion of PFOA to 5:3 FTCA with a 1:1 stoichiometry, and a measured fluoride concentration of 10 mg L^-1^ (1) The initial PFOA concentration corresponds to 0.2415 mM. (2) Assuming 60% conversion, 0.1449 mM 5:3 FTCA is formed. (3) Because PFOA contains 15 F atoms and 5:3 FTCA contains 11, the assumed 1:1 conversion release four F atoms per transformed PFOA molecule, corresponding to a theoretical fluoride release of 0.5796 mM. (4) The assumed measured fluoride concentration of 10 mg L^-1^ corresponds to 0.5263 mM F^−^. (5) This represents 90.8% recovery of the theoretical fluoride expected from the transformed PFOA fraction. (6) Relative to the total initial fluorine, 40.0% remains in residual PFOA, 44.0% is retained in 5:3 FTCA, and 14.5% is recovered as measured F^−^, leaving approximately 1.5% unaccounted for because of rounding and the illustrative assumptions. Thus, parent-PFAS transformation does not necessarily indicate extensive defluorination, highlighting the need for direct fluoride measurements and fluorine mass balance when evaluating PFAS biodegradation and claims of destruction. These percentages are illustrative calculations based on the stated assumptions and should not be interpreted as experimentally measured fluorine-fate data.

Non-biological treatment studies provide a useful comparator for the same analytical distinction. Co(II) phthalocyanine with Ti(III) citrate achieved 10.9% defluorination of technical PFOS and 54.5% of branched PFOS isomers after 8 h, whereas vitamin B12 achieved only 2.4% defluorination (Mirabediny et al., 2024). Similarly, cold atmospheric plasma produced 99.99% PFOS degradation but only 35% defluorination, with stepwise transformation products and fluorine mass-balance recoveries of 31%–106% (Kumar et al., 2026). Although these systems are not evidence of microbial activity, they reinforce the need to quantify degradation and defluorination separately, a principle equally relevant to biological remediation.

A fluorine mass balance should link the initial fluorine inventory to released F^−^ and residual fluorine in parent PFAS, identified and unidentified transformation products, biomass, particulate phases, and other relevant compartments. Targeted LC–MS/MS should be complemented by TOF, EOF, AOF, HRMS, nontargeted analysis, and transformation-product libraries to capture organofluorine beyond the targeted analyte list (Forster et al., 2023; Idjaton et al., 2024; Liu et al., 2025). TOF, EOF, and AOF represent operationally defined fluorine pools determined by different analytical procedures. Their measured values are method-dependent and should not be treated as directly interchangeable or summed, because the pools may overlap and their recoveries may differ. Mass-balance closure should be interpreted in light of analytical recovery, measurement uncertainty, and potentially unmeasured fluorine pools. Carbon-product analysis is additionally required to support mineralisation claims.

## 3 How strong is the evidence for microbial PFAS biodegradation?

The literature demonstrates that microorganisms can transform selected PFAS and that microbial C–F cleavage is chemically achievable, although the strength of evidence varies substantially. A meta-analysis of 97 microbial PFAS biotransformation studies found that transformation was more likely under aerobic conditions, in defined or axenic cultures, at higher PFAS concentrations, and for compounds containing fewer fluorine atoms. Activity also depended on chain length, branching, and headgroup. Anaerobic systems with defined electron donors and acceptors, identified organisms or enzymes, and comprehensive transformation-product data remain comparatively underrepresented (Skinner et al., 2025). Cross-study comparisons must therefore account for both the reported endpoint and experimental conditions. Results from defined systems or elevated PFAS concentrations may not translate directly to environmental settings, where matrix properties affect partitioning, bioavailability, microbial activity, and analytical recovery.

Parent depletion is the most frequently reported observation, but microbial systems may combine adsorption, accumulation, transformation, and abiotic redistribution. Saifur et al. (2026) reported 10.9%–38.3% adsorption without parent-PFAS transformation in anaerobic wastewater cultures over 90 days. In aerobic cultures after 21 days, adsorption ranged from 26.9% ± 1.2% to 55.8% ± 1.4%, and biotic accumulation was 13.35%–17.55%. Transformation was reported for PFOA (47.87% ± 1.6%), PFOS (28.96% ± 0.6%), and 6:2 FTS (43.1% ± 1.0%), with shorter-chain products and limited defluorination. These concurrent processes show why single-phase measurements may not represent the overall PFAS mass balance. Representative studies and their evidence limitations are summarised in Table 1.

**TABLE 1:** Evidence for microbial PFAS biotransformation and defluorination.

Study	PFAS/ substrate	Biological system and conditions	Parent removal or transformation	Transformation products	Direct evidence of defluorination	Evidence-based interpretation
Kwon et al. (2014)	PFOS	*Pseudomonas aeruginosa* HJ4; 30 °C–37 °C; 48 h	Approximately 67% biological decomposition reported	Not reported	Fluoride release not reported	Parent-PFOS loss was reported; the extent of C–F cleavage cannot be established from the reported evidence
Yi et al. (2016)	PFOA	*Pseudomonas parafulva* YAB1; 30 °C, pH 7; 96 h	32.4% degradation; 48.1% with glucose	Not reported	Fluoride release not reported	Parent-PFOA loss was reported; transformation pathways and defluorination remain unconfirmed by the supplied evidence
Wang et al. (2011)	6:2 FTS	Aerobic activated sludge from three wastewater treatment plants; 90 days	36.3% parent decrease; observed products accounted for 6.3% of the initial amount	5:3 acid, PFBA, PFPeA, PFHxA, 5:2 ketone, 5:2 sFTOH and intermediates	Fluoride release not reported	Transformation products demonstrate biotransformation, but the incomplete mass balance does not establish the fate of unaccounted material
Zhang et al. (2013)	6:2 and 8:2 FTOH	Anaerobic methanogenic digester sludge; up to 181 days	Transformation to polyfluorinated acids and related products	FTCA, FTUCA, 5:3 acid, 7:3 acid; trace PFHxA and PFOA	Fluoride release not reported	Precursor transformation was demonstrated; the reported products do not establish extensive defluorination
Wang et al. (2012)	5:3 and 7:3 fluorinated acids	Aerobic activated sludge; 90 days	Limited conversion of 7:3 acid; multiple products from 5:3 acid	4:3 acid, 3:3 acid, PFPeA, PFBA and intermediates	Fluoride release not reported	One-carbon-removal pathways were proposed, but the evidence does not establish complete carbon mineralisation or extensive defluorination
Kim et al. (2012)	4:2, 6:2 and 8:2 FTOH	*Pseudomonas butanovora* and *Pseudomonas oleovorans*	Biotransformation reported for all three FTOHs; greater defluorination of 4:2 FTOH	Shorter-chain ketones, sFTOHs, PFCAs and polyfluorinated acids	Defluorination reported; quantitative fluoride values and fluorine balance not available	Direct evidence of defluorination was reported, with the extent dependent on substrate
Chetverikov et al. (2017)	PFOS	*Pseudomonas plecoglossicida* 2.4-D; liquid culture and soil	Complete PFOS utilisation reported in liquid culture after 6 days; 75% degradation in soil	PFHpA	Free fluoride ions released	Fluoride release supports defluorination; the overall fluorine balance and extent of fluorine removal remain unresolved
Yang et al. (2022)	6:2 FTSA	*Rhodococcus jostii* RHA1; sulfur-dependent conditions	Desulfonation and transformation were condition-dependent	Four confirmed metabolites, one tentative metabolite; PFHpA reported as a new metabolite	Defluorination occurred under sulfur-free conditions following desulfonation; not observed for 6:2 FTSA in sulfur-rich medium	Demonstrates condition-dependent defluorination and a proposed enzymatic pathway; complete fluorine fate was not established
Chiriac et al. (2023)	PFOA and PFOS	*Pseudomonas aeruginosa* and *Pseudomonas putida*; 96 h	PFOA: 19.0%–27.9%; PFOS: 46.9%–47.3% transformation reported	Shorter-chain products reported; specific product assignments require verification against the full article	Fluoride release not reported	Transformation was reported, but the evidence does not establish the extent of defluorination
Wijayahena et al. (2025)	PFOS, 6:2 FTS and 5:3 FTCA	Aerobic *Labrys portucalensis* F11; up to 194 days	PFOS: 90% at 100 days and 96% at 194 days; 6:2 FTS: 21% decrease at 100 days; 5:3 FTCA: 58% decrease at 100 days	Multiple shorter-chain products; three defluorinated PFOS metabolites identified	Fluoride increase and defluorinated metabolites reported; overall fluorine mass balance not available	Defluorination is supported, but parent-PFOS removal must not be interpreted as an equivalent percentage of defluorination or complete destruction
Saifur et al. (2026)	PFOA, PFOS, PFBS, 6:2 FTS and GenX	Aerobic activated/nitrification sludge; anaerobic digestate and KB-1 culture	Aerobic: PFOA 47.87%, PFOS 28.96% and 6:2 FTS 43.1% transformation. Anaerobic: no parent transformation over 90 days	Shorter-chain carboxylates and sulfonates	Limited defluorination reported; quantitative fluoride and fluorine mass-balance data not specified	Separates biological transformation from adsorption and accumulation; evidence indicates limited defluorination rather than complete destruction

Abbreviations: FTOH, fluorotelomer alcohol; FTSA, fluorotelomer sulfonic acid; FTS, fluorotelomer sulfonate; PFAS, per- and polyfluoroalkyl substances; PFBS, perfluorobutane sulfonate; PFOA, perfluorooctanoic acid; PFOS, perfluorooctanesulfonate; PFHpA, perfluoroheptanoic acid.

“Not reported” indicates that the relevant evidence was not specified in the study information available for this table; it does not establish that the process did not occur. Parent removal, biotransformation, defluorination and mineralisation are distinct outcomes. Fluoride release supports defluorination, but claims of extensive fluorine removal require a defensible fluorine mass balance. Fluoride release alone does not demonstrate carbon mineralisation.

Matrix effects are critical in wastewater, biosolids, soil, and sediment. In wastewater and biosolids, partitioning between water and solids can make apparent aqueous removal difficult to distinguish from transfer to sludge; biosolids composition and microbial weathering may also alter PFAS partitioning over time (Lewis et al., 2023). In soil, retention and leaching depend on soil properties and application conditions. Field-relevant lysimeter experiments have demonstrated differences in short- and long-chain PFAS distribution between soil and leachate following biosolids application (Peter et al., 2025), while the delivery matrix can influence PFAS mobility and bioavailability (Sidhu et al., 2026). Studies should therefore assess aqueous and solid-associated PFAS, where relevant, and account for changing partitioning during incubation. Groundwater assessments should also consider sorption to aquifer materials and the accumulation of transformation products.

Transformation-product analysis provides stronger mechanistic evidence but remains incomplete when restricted to conventional target lists. Liu et al. (2025) identified biotransformation products of 27 PFAS involving glucuronidation, sulfation, dealkylation, oxidation, and related reactions, incorporating them into nontargeted HRMS libraries; some predicted products raised potential developmental or mutagenic concerns. Bulk fluorine methods can reveal additional fluorine pools. Forster et al. (2023) found that TOF methods captured up to 99% more organic fluorine than targeted LC–MS/MS across samples containing 39 PFAS, including GenX and PFBS. AOF recoveries were 46%–112% for individual PFAS and 87% for mixtures, while EOF recoveries were 72%–99% and 91%, respectively. Idjaton et al. (2024) found that AOF and EOF represented 1%–23% and 0.1%–2% of TOF, respectively; targeted PFAS accounted for only 0.2%–11% of AOF and 0.003%–5% of EOF. These differences reflect the operationally defined nature of these measurements: TOF, EOF, and AOF represent different, potentially overlapping fluorine pools and should not be treated as interchangeable or additive. Bulk fluorine measurements reveal analytical blind spots but cannot identify the chemical species present. Results should therefore be interpreted using method-specific recovery and uncertainty. In complex matrices, recovery should be validated for each sample type because extraction losses, background fluorine, and phase partitioning can affect estimates of defluorination. Fluoride measurements should include appropriate blanks, background correction, and matrix-matched calibration or spike-recovery assessment.

A rigorous investigation should combine parent-PFAS measurements with comprehensive transformation-product analysis, direct F^−^ quantification, fluorine mass balance, and appropriate sterile, killed-cell, sorbent, matrix, substrate-free, and uninoculated controls. Where feasible, ^13^C-labelled PFAS can help trace PFAS-derived carbon and identify carbon-containing transformation products. However, attribution of released fluoride should rely primarily on background correction, appropriate controls, and fluorine mass balance rather than ^13^C tracing alone. Long-term monitoring is also essential because transformation products can accumulate after apparent parent removal. The 664-day WBC-2 bioelectrochemical experiment is an instructive example: although >99.0% PFOS removal occurred within 21 days in defined water, and >98.9% removal of PFOS, PFOA, PFHxA, and PFHxS occurred in contaminated groundwater after 102 days at −450 mV versus Ag/AgCl, PFOA, PFPrA, and PFBA accumulated following repeated PFOS additions. PFOA represented <1.8% of the total spiked PFOS mass (Yang et al., 2026). Background-corrected fluoride release and limited transformation in controls supported reductive microbial transformation and defluorination, but the long-term product profile demonstrates why removal alone is insufficient. This study illustrates the value of testing beyond defined aqueous systems, although further work is needed across groundwater chemistries and contaminant concentrations.

The evidence hierarchy should be applied consistently under environmentally relevant conditions, with sampling and analytical methods adapted to each matrix. Parent depletion and product identification establish initial changes, while direct fluoride measurement and fluorine-fate analysis are needed to quantify defluorination. Carbon-product analysis addresses the separate question of mineralisation. Defluorination and detoxification must also be evaluated as distinct outcomes. Although fluoride release provides evidence of C–F bond cleavage, it does not demonstrate that the resulting products are less toxic or environmentally benign. Detoxification requires separate assessment of transformation-product persistence, mobility, and toxicity using appropriate chemical and biological endpoints (Liu et al., 2025; Verma et al., 2026). Environmental studies should assess relevant aqueous and solid phases, while groundwater studies should account for background fluoride and potential sorption to aquifer materials. Fluoride recovery and fluorine-mass-balance closure should be reported alongside analytical recovery and uncertainty rather than assumed from results in defined media. Biotransformation studies remain valuable even when defluorination has not been demonstrated, as they can reveal susceptible structures, transformation pathways, and candidate enzymes. The key research gap is quantitatively linking transformation-product formation to fluorine fate at environmentally relevant concentrations and in complex matrices. Such studies should complement controlled laboratory experiments that establish mechanisms and support reliable mass-balance interpretation.

## 4 From evidence to engineering: designing microbial systems for true PFAS remediation

The evidence hierarchy should directly shape biological engineering objectives. Rather than selecting organisms solely for PFAS tolerance or parent-compound disappearance, remediation systems should target the specific outcome required, whether biological transformation, C-F bond cleavage, extensive defluorination, or mineralisation. This requires integration of microbial physiology with enzyme engineering, metabolic pathway design, and process control. The objective should be to distinguish systems that transform PFAS from those that demonstrably remove organofluorine, while evaluating carbon fate separately when mineralisation is claimed. Importantly, the engineering strategies discussed below differ in their level of technological maturity: some biological systems have demonstrated PFAS transformation or defluorination, whereas several proposed approaches remain prospective and require PFAS-specific experimental validation.

### 4.1 Engineering microbes for C-F bond cleavage

Several engineering approaches including metabolic engineering, directed evolution, synthetic biology, CRISPR-based pathway reconstruction, metabolic flux analysis, and co-metabolic design that could potentially be used to develop microbial systems capable of PFAS transformation or defluorination. However, these approaches should be regarded as future engineering opportunities unless PFAS-specific activity has been experimentally demonstrated (Olawade et al., 2025). In particular, the successful application of CRISPR, directed evolution, or pathway reconstruction to other biological systems does not, by itself, establish their effectiveness for engineering PFAS-defluorinating microorganisms.

Enzymes capable of cleaving C–F bonds in selected fluorinated compounds provide potential starting points for enzyme discovery and engineering. Reported enzyme activities include those associated with hydrolases, reductases, lyases, and oxygenases, although activity against a model organofluorine substrate cannot be assumed to extend to structurally diverse PFAS (Natarajan et al., 2005; Wackett, 2025). Because PFAS persistence reflects both chemical stability and biological constraints, mechanism-guided optimisation of existing defluorinating activities is a potential strategy, but its applicability to environmentally relevant PFAS requires direct validation (Wackett, 2024; 2025).

Recent work identified gut microbial defluorinases and used computational and protein-engineering approaches to transfer and predict defluorination activity, achieving up to 95% prediction accuracy for full-length sequences (Probst et al., 2025). Similarly, a multifunctional hydrolase from *Serratia liquefaciens* IMD717 defluorinated monofluoroethylglycine and fluoroacetate, while Ser101 mutation altered dehalogenase activity, demonstrating the potential of active-site engineering for C-F cleavage (Khan et al., 2026). These findings establish relevant proof-of-concept for defluorination and enzyme engineering, but they should not be interpreted as evidence of PFAS defluorination unless activity against PFAS substrates has been demonstrated. Together, these advances support a transition from enzyme discovery toward mechanism-guided defluorinase optimisation, with PFAS substrate scope, catalytic activity, product identity, and fluoride release serving as essential validation criteria.

PFAS structural diversity also favours the future development of engineered reaction networks, rather than reliance on a single organism or enzyme. Such networks could combine oxidative, reductive, hydrolytic, and dealkylation reactions to process structures that may be inaccessible to individual catalysts (Jin et al., 2023; Che et al., 2025). However, the proposed integration of these reactions into a coordinated PFAS-degrading network remains an engineering objective; the feasibility of combining individual activities must be established experimentally, including assessment of intermediate accumulation and fluorine fate.

The sequential photocatalysis–fungal treatment of PFOA illustrates the potential complementarity of physicochemical and biological processes, whereas the WBC-2 bioelectrochemical system provides a biological example of long-term microbial transformation and measurable defluorination (Khan et al., 2023b; Yang et al., 2026). These examples provide evidence for specific treatment configurations and biological activities, but do not establish the performance of engineered microbial consortia or integrated treatment trains as a general solution for PFAS remediation. Such systems could be developed into treatment trains in which separation concentrates PFAS, physicochemical processes modify recalcitrant structures, and selected microbial or enzymatic systems are used to transform characterised intermediates. The compatibility, sequencing, and overall performance of these treatment stages would require validation using fluorine mass balances and relevant environmental matrices.

Fluoride generated during C–F cleavage can inhibit microbial growth and activity, requiring consideration of fluoride tolerance, transport, sequestration, and product resistance alongside catalytic optimisation (Wackett, 2024; 2025; Wackett and Robinson, 2024). Scale-up remains constrained by matrix complexity, competing compounds, microbial stability, reactor configuration, energy and cost requirements, secondary pollution, and process control, while GEM-based systems additionally require containment, ecological risk assessment, reactor safety, and economic evaluation (Tshangana et al., 2025; Sanzana et al., 2025; Olawade et al., 2025). Thus, the engineering endpoint should be reproducible PFAS transformation or biodefluorination, as appropriate to the intended application, with claims of destructive remediation supported by quantitative fluorine-fate evidence, rather than maximum parent-PFAS removal under ideal laboratory conditions. The distinction between demonstrated performance and proposed engineering capability should be maintained when interpreting laboratory studies and assessing readiness for practical application.

### 4.2 A verification framework for engineered PFAS-Degrading systems

Engineering C–F-cleaving activity must be accompanied by verification capable of distinguishing genuine defluorination from parent-PFAS disappearance, redistribution, or incomplete transformation. A practical verification workflow should quantify parent PFAS and transformation products, measure background-corrected fluoride release, and assess fluorine mass balance. Che et al. (2025) provide a quantitative basis for assessing defluorination from fluoride released relative to PFAS removed and the fluorine content of the substrate. Targeted LC–MS/MS, HRMS and nontargeted analysis, together with TOF, EOF, and AOF measurements, should be used complementarily because targeted analyses may overlook unknown transformation products and organic-fluorine pools (Forster et al., 2023; Idjaton et al., 2024; Khan, 2026b). TOF, EOF, and AOF are operationally defined measurements of different, potentially overlapping fluorine pools; their results should not be treated as directly interchangeable or summed. A fluorine mass balance should account, where analytically feasible, for released F^−^, residual parent PFAS, identified transformation products, and other measurable organic-fluorine pools (Kumar et al., 2026; Verma et al., 2026). The degree of mass-balance closure should be interpreted in light of analytical recovery, measurement uncertainty, and the possibility of unmeasured or unrecovered fluorine.

For mineralisation claims, carbon-product analysis must establish conversion of PFAS-derived carbon to inorganic carbon, such as CO_2_ or dissolved inorganic carbon, rather than its persistence in organic intermediates. Where feasible, ^13^C-labelled PFAS can help distinguish PFAS-derived inorganic carbon from background carbon. The persistence and toxicity of transformation products should also be evaluated because shorter-chain PFAS and other intermediates can remain persistent and mobile or exhibit distinct toxicological properties (Liu et al., 2025; Verma et al., 2026; Dai and Khan, 2026). Sterile, killed-cell, uninoculated, sorbent, matrix, and substrate-free controls are required to distinguish biological activity from abiotic reactions, adsorption, precipitation, and phase redistribution, while replication and long-term monitoring strengthen evidence for reproducible and sustained PFAS transformation. These measurements should be reported alongside experimental conditions, analytical recoveries, and uncertainty so that engineered systems can be compared on a common evidential basis (Huang et al., 2025; Yang et al., 2026). For proposed engineered systems, this verification should be applied at the outset of development, so that evidence of PFAS transformation or defluorination is established independently of improvements in parent-compound removal.

Engineered systems should therefore be evaluated against the specific outcome they demonstrate, rather than a general label of “PFAS degradation”. Parent depletion, verified transformation products, fluoride release, and carbon conversion provide complementary but non-equivalent measures. Detoxification should be treated as an additional, independent endpoint: even extensive defluorination may yield transformation products that remain persistent, mobile, or toxic. Accordingly, claims of detoxification require direct assessment of transformation-product hazards and cannot be inferred from fluoride release, parent-PFAS depletion, or carbon mineralisation alone (Verma et al., 2026). This approach aligns engineering objectives with measurable chemical outcomes and reduces the risk of selecting systems that remove PFAS from solution but leave persistent organofluorine products. It also provides a clear distinction between current proof-of-concept findings and future engineering proposals, ensuring that claims of PFAS-specific capability are proportionate to the available evidence.

## 5 Toward a more rigorous definition of PFAS biodegradation

Terminology matters because it shapes remediation evaluation, environmental-risk interpretation, and the biological traits selected for engineering. A statement such as “90% PFAS degradation” may imply substantial environmental benefit even when most fluorine remains in transformation products. The distinction between separation and destruction is already recognised in PFAS treatment science and should be applied equally to biological studies (Meegoda et al., 2022). The central issue is therefore not whether a single term can encompass all biological changes, but whether reported terminology accurately reflects the measured endpoint. This distinction is particularly important because PFAS susceptibility depends strongly on chain length, branching, headgroup, fluorination pattern, ether functionality, precursor structure, and environmental conditions (Skinner et al., 2025; Che et al., 2025).

Overstated biodegradation claims can misdirect discovery and engineering by favouring organisms that rapidly remove PFAS from solution through sorption or precursor transformation rather than C–F cleavage. Screening directly for fluoride release instead targets the biochemical function required for destructive remediation, while high-throughput defluorination assays, sequence-function prediction, directed evolution, and enzyme engineering provide routes to selecting and optimising appropriate biological systems (Simon et al., 2025; Probst et al., 2025; Wackett, 2025). Accordingly, the engineering metric should shift from maximum parent-PFAS removal towards quantified defluorination, fluorine-fate accounting, and minimisation of persistent intermediate accumulation. Standardisation is essential to support this transition. A weight-of-evidence framework analogous to PFAS conceptual site-model approaches can integrate PFAS source, fate, transport, chemical families, and tiered analytical evidence (Gamlin et al., 2024). For biodegradation studies, this should include parent depletion, transformation products, fluoride release, residual organofluorine, analytical recovery, appropriate controls, and, where relevant, carbon fate.

Biological treatment need not independently achieve complete mineralisation. Given PFAS structural diversity and current scale-up constraints, engineered microbial systems may be most effective within integrated treatment trains combining separation, physicochemical transformation, and biological processing of susceptible intermediates (Meegoda et al., 2022; Tshangana et al., 2025; Sanzana et al., 2025). The same mechanistic knowledge could support benign-by-design and safe-and-sustainable-by-design strategies by identifying fluorinated structures more amenable to biological C–F cleavage (Che et al., 2025). The proposed framework is intended to make claims more precise, not to discount studies that demonstrate biotransformation without defluorination. Such studies remain valuable for identifying pathways, intermediates, and candidate enzymes, but their environmental significance depends on the persistence, mobility, and toxicity of the products formed. The field should consequently move from reporting parent-compound disappearance alone towards endpoint-specific, quantitatively supported assessments of PFAS removal, biotransformation, biodefluorination, and mineralisation.
